# Differences in the Prevalence and Clinical Correlates Between Early-Onset and Late-Onset Major Depressive Disorder Patients with Comorbid Abnormal Lipid Metabolism

**DOI:** 10.3390/metabo15020117

**Published:** 2025-02-11

**Authors:** Xiao Huang, Anshi Wu, Xiangyang Zhang

**Affiliations:** 1 Department of Anesthesiology, Beijing Chao-Yang Hospital, Capital Medical University, 8 Workers Stadium South Road, Chaoyang District, Beijing 100020, China; huanghuang94@yeah.net; 2 Hefei Fourth People’s Hospital, Hefei 230022, China; 3 Affiliated Mental Health Center, Anhui Medical University, 316 Huangshan Road, Shushan District, Hefei 230022, China

**Keywords:** major depressive disorder, abnormal lipid metabolism, age onset, outpatients

## Abstract

Background and Objectives: Growing evidence suggested that abnormal lipid metabolism (ALM) was associated with an increased severity of depressive symptoms, but no previous studies have examined the differences in comorbid ALM in major depressive disorder (MDD) patients of different ages of onset. We aim to compare the differences in the prevalence and clinical correlates of ALM between early-onset and late-onset patients with first-episode and drug-naive (FEDN) MDD patients. Methods: Using a cross-sectional design, we recruited a total of 1718 FEDN MDD outpatients in this study. We used the 17-item Hamilton Rating Scale for Depression (HAMD-17), The Hamilton Anxiety Rating Scale (HAMA), the Positive and Negative Syndrome Scale (PANSS) positive subscale, and Clinical Global Impression-Severity Scale (CGI-S) to assess their depression, anxiety, and psychotic symptoms and clinical severity, respectively. Results: There were 349 patients (20.3%) in the early-onset subgroup and 1369 (79.7%) in the late-onset subgroup. In this study, 65.1% (1188/1718) of patients were diagnosed with ALM. The prevalence of ALM in the late-onset group (81.5%, 1116/1369) was significantly higher than that in the early-onset group (20.6%, 72/349) (*p* = 0.36, OR = 1.147, 95%CI = 0.855–1.537). The HAMD total score (OR = 1.34, 95% CI = 1.18–1.53, *p* < 0.001) was the only risk factor for ALM in early-onset MDD patients. In late-onset MDD patients, the HAMD total score (OR = 1.19, 95% CI = 1.11–1.28, *p* < 0.001), TSH (OR = 1.25, 95% CI = 1.16–1.36, *p* < 0.001), CGI (OR = 1.7, 95% CI = 1.31–2.19, *p* < 0.001), and anxiety (OR = 2.22, 95% CI = 1.23–4.02, *p* = 0.008) were risk factors for ALM. Conclusion and Scientific Significance: Our results suggest that there are significant differences in the prevalence and clinical factors of comorbid ALM between early-onset and late-onset FEND MDD patients.

## 1. Introduction

Major depressive disorder (MDD) remains one of the most burdensome psychiatric disorders in the world, severely impairing an individual’s daily functioning and quality of life and causing significant disability worldwide [1,2]. The pathogenesis of MDD is currently unclear, but chronic inflammatory responses, inflammatory vesicle activation, neurotransmitter alterations and deregulation, oxidative stress, and mitochondrial dysfunction are all involved [3,4]. Furthermore, the identification of clinical correlates has great potential for the prevention of the onset and development of MDD.

Major depressive disorder (MDD) is a highly heterogeneous condition, with early-onset and late-onset being significant subtypes. Research has highlighted key differences between early- and late-onset MDD as well as other neurodegenerative diseases or mood disorders [5,6]. For instance, Rozing et al. found a stronger correlation between low-grade inflammation and late-onset depression compared to early-onset depression [3]. Similarly, in postpartum depression, early-onset and late-onset cases follow distinct patterns [7]. Mai et al. reported differences in brain activity between early- and late-onset depression, revealing that early-onset patients had lower modularity in brain activity compared to late-onset patients [8]. These findings suggest that early- and late-onset depression may involve different pathological changes and biological mechanisms. In addition to these neurobiological differences, early-onset mental illness is often linked to a more chronic and severe clinical course, with a higher rate of co-occurring psychiatric disorders [9,10]. Patients with early-onset MDD also tend to experience longer disease durations and exhibit higher neuroticism scores [11]. Unlike late-onset depression, early-onset MDD is more likely to present with irritability rather than pronounced sadness, and the treatment approaches for late-onset MDD may not be suitable for early-onset patients [12]. While both subtypes share clinical symptoms and have identical diagnostic criteria, the recurrence rate of early-onset MDD is higher. These distinctions highlight the importance of separating the studies of early-onset and late-onset MDD.

Recently, researchers have focused on the correlation between abnormal lipid metabolism (ALM) and MDD. Many studies have focused on the relationship between depression and dyslipidemia, showing that patients with MDD tend to have higher lipid levels than the general population. For example, low high-density lipoprotein cholesterol (HDL-C) (<40 mg/dL) levels in MDD patients are significantly associated with suicide rates in patients with MDD [13]. Gohar et al. revealed that TC and LDL-C concentrations were significantly associated with depression [14]. A study by Korczak et al. showed that cardiovascular disease risk factors were common in children and adolescents with MDD. They reported that 25% of the participants were in the overweight/obese range and 28% had increased TC levels. Overweight/obese participants had higher concentrations of non-HDL cholesterol compared with healthy-weight participants [15]. Significant reductions in reverse cholesterol transport (RCT) (mainly lower HDL cholesterol and paraoxonase 1) and increases in lipid peroxidation and aldehyde formation were found in patients with MDD and bipolar disorder (BD) compared to healthy controls [16], indicating that the lowering of RCT is a critical antioxidant and anti-inflammatory route in MDD/BD [17,18]. At the same time, antidepressant medication reduces the levels of HDL-C, LDL-C, and TC [19], suggesting that physicians should consider the role of lipids in planning and managing therapeutic strategies for patients with MDD [20].

Several molecular mechanisms have been proposed to explain the connection between lipids and mood disorders. One hypothesis suggests that low cholesterol levels may be linked to reduced serotonin uptake and changes in the viscosity of brain cell membranes, with evidence showing that lipid fluidity plays a key role in regulating serotonin binding in mouse brain membranes [21]. Due to its high lipid content and limited antioxidant defenses, the brain is particularly vulnerable to oxidative stress [22]. Another theory suggests that metabolic syndrome (MetS), which is marked by increased lipid peroxidation and oxidative stress, may contribute to the co-occurrence of anxiety and depression through shared pathways involving reactive oxygen and nitrogen species (RONS) and nitro-oxidative stress [17]. Additionally, it is hypothesized that lower esterification of serum cholesterol could increase the susceptibility to depression by altering cell membrane microviscosity [18]. While some studies suggest that ALM is associated with more severe depressive symptoms, no research has yet examined how comorbid ALM affects MDD patients with different ages of onset.

Previous studies have explored the relationship between the age of onset in MDD and comorbid ALM, highlighting the importance of both factors in the progression of MDD. However, no research has specifically examined ALM across different age-onset subgroups of MDD. To our best knowledge, this is the first study to compare ALM differences between early-onset and late-onset first-episode, drug-naïve (FEDN) MDD patients in a large sample. We aimed to identify the clinical and metabolic factors associated with ALM in early- and late-onset MDD patients, respectively. This study will enhance our understanding of MDD subtypes, inform risk stratification, and contribute to the development of targeted therapeutic strategies and potential biomarkers.

## 2. Methods

### 2.1. Subjects

A total of 1718 patients were enrolled from the First Hospital of Shanxi Medical University in Shanxi Province, China. The study was conducted from 2016 to 2017. All participants gave written informed consent before entering the trial. The study was approved by the ethics committee of the First Hospital of Shanxi Medical University (ID number: 2016-Y27).

Inclusion criteria were (1) Han Chinese patients between the ages of 18 and 60; (2) those meeting the diagnostic requirements of the Structure Clinical Interview for DSM-IV (SCID), which was conducted by two qualified clinical psychiatrists; and (3) patients in the first episode who were not taking medication.

Exclusion criteria were (1) having a serious physical illness; (2) being pregnant or breastfeeding women; (3) alcohol or drug abuse and dependence, except for nicotine; (4) another psychiatric disorder diagnosis on Axis I; and (5) being unable to understand and provide signed informed consent.

In this study, 22 was used as the cutoff value for early vs. late onset in MDD patients (early onset, <22 years; late onset, ≥22 years) [23,24,25].

### 2.2. Demographic Characteristics, Clinical Interview, and Assessment

The 17-item Hamilton Depression Rating Scale (HAMD) was used to measure the severity of depression [26]. The scale includes eight five-point items ranging from 0 to 4 and nine three-point items ranging from 0 to 2. These values were summed to indicate the severity of depression as none (<6), mild (6–13), moderate (14–18), severe (19–23), or very severe (≥24) [27].

The Hamilton Anxiety Rating Scale (HAMA) was used to assess the severity of anxiety [28]. The scale consists of 14 items, each of which provides five alternative responses to honest feelings, ranging from 0 to 4 [29]. In this study, patients were considered to have severe anxiety symptoms when they scored more than 24.

The Positive and Negative Syndrome Scale (PANSS) positive subscale was applied to assess psychotic symptoms [30]. Each item has a score range of 1–7, with a total score of 7–49 [31]. Our study used 15 as the cutoff of the PANSS-positive symptom.

The Clinical Global Impression-Severity (CGI-S) scale was used to assess the severity of the illness. A CGI-S score ranges from 1 (not at all) to 7 (the most severe illness) [32].

Demographic information for this study consisted of age, gender, marital status, years of education, and duration of illness. In this study, all demographic and clinical data were collected by two professional researchers. We used the Chinese versions of these scales. The results of repeated evaluation after training indicated that the correlation coefficients between them were >0.8.

### 2.3. Biomarker Measurements

Fasting venous blood was collected from 6:00 to 8:00 a.m. in all enrolled patients. Plasma TC, TG, HDL-C, LDL-C, and blood glucose concentrations were measured in the diagnostic laboratory of the first clinical medical college, Shanxi Medical University. Thyroid peroxidase antibody (A-TPO), anti-thyroglobulin (A-TG), thyroid-stimulating hormone (TSH), free thyroxine (FT4), and free triiodothyronine (FT3) were detected using a Roche C600 Electrochemiluminescence Immunoassay Analyzer (Roche Dianostics, Indianplis, IN, USA). In this study, ALM was diagnosed as one of the following criteria: high TC was defined as TC ≥ 200 mg/dL (5.20 mmol/L); high TG as TG ≥ 150 mg/dL (1.70 mmol/L); high LDL-C as LDL-C ≥ 130 mg/dL (3.40 mmol/L); and low HDL-C as HDL-C ≤ 40 mg/dL (1.00 mmol/L) [33].

### 2.4. Statistical Analysis

All statistical analyses were performed using IBM SPSS 25.0. Sociodemographic and clinical data were compared between the subgroups with and without ALM in early-onset and late-onset MDD patients by using analysis of variance (ANOVA) and chi-square tests for continuous and categorical variables, respectively. Nonparametric tests were used to compare non-normally distributed variables. Bonferroni correction was used to adjust for multiple tests. We investigate the gender difference in the prevalence of ALM in both the early-onset and late-onset groups. Additionally, a binary logistic regression analysis was conducted to evaluate which factors were most strongly correlated with ALM in early- and late-onset MDD patients. We used the area under the receiver operating characteristic (AUCROC) curve to determine the discriminatory power of important variables between patients with and without ALM in early-onset and late-onset MDD patients, respectively. A consistency statistic > 0.7 is generally considered acceptable [34,35]. We also combined parameters with AUC values ≥ 0.7 to distinguish patients with ALM from those without ALM. A *p*-value of 0.05 was considered as statistical significance.

## 3. Results

### 3.1. Prevalence of ALM in Early-Onset and Late-Onset MDD Patients

According to the cutoff value of 22 years of age at onset, there were 349 patients (20.3%) in the early-onset subgroup and 1369 (79.7%) in the late-onset subgroup. A total of 588 men and 1130 women were included in this study. The incidence of early-onset MDD in men (149/200, 42.7%) was significantly higher than the incidence of late-onset in men (439/930, 32.1%) (χ^2^ = 13.949, *p* < 0.001, OR = 1.6). In this study, 65.1% (1188/1718) of patients were diagnosed with ALM. The prevalence of ALM in the late-onset group (81.5%, 1116/1369) was significantly higher than that in the early-onset group (20.6%, 72/349) (*p* = 0.36, OR = 1.147, 95% CI = 0.855–1.537).

### 3.2. Comparison of Demographic and Clinical Variables and Lipid Levels Between ALM and Non-ALM Subgroups in Early- and Late-Onset MDD Patients

Table 1 shows that in late-onset patients, MDD patients combined with ALM had a longer duration of disease (F = −3.949, *p* < 0.001), a greater age of onset (F = −2.03, *p* = 0.042) and higher BMI (F = 10.739, *p* = 0.001). No significant differences were seen in early-onset MDD patients.

Table 2 shows that among patients with early- and late-onset MDD, those with ALM had higher HAMD, HAMA, PANSS positive subscale, CGI scores, TSH levels, SBP, and DBP (all *p* < 0.05). Patients with ALM had a greater rate of psychotic symptoms compared to those without ALM (*p* < 0.05). Only in patients with late-onset MDD, the subgroup with ALM had higher rates of suicide attempts and anxiety, higher age, age at onset, duration of disease, blood glucose, and ATG levels (all *p* < 0.05). However, in patients with early-onset MDD, significant differences in psychotic symptoms, HAMA scores, and DBP were not corrected using Bonferroni’s method (*p* ≥ 0.05/19). In the late-onset group, the significance of age differences, age at onset, suicide attempts, and anxiety were not corrected using Bonferroni’s method (*p* ≥ 0.05/19). No gender difference was found in ALM in both the early-onset and late-onset subgroups.

### 3.3. Risk Factors Associated with ALM in the Early-Onset Group

**Figure 1:**
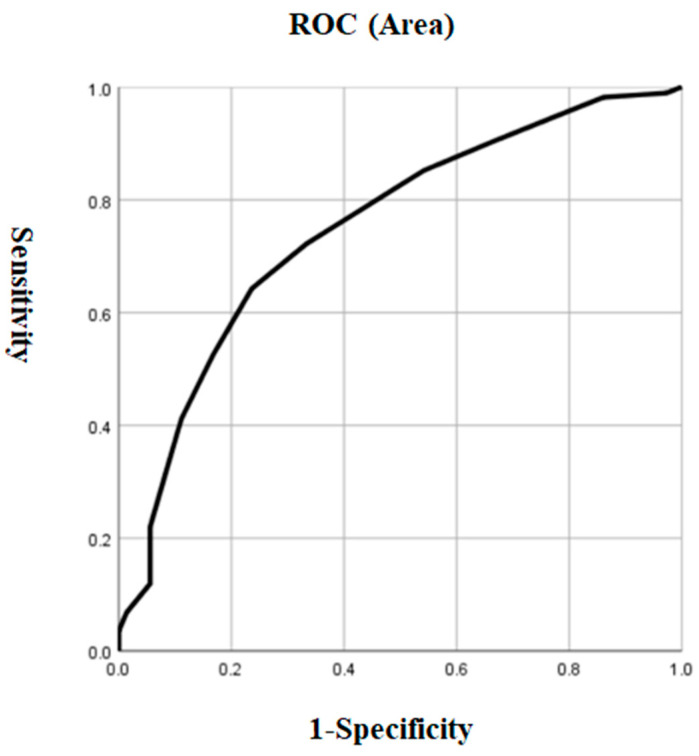
The discriminatory capacity of HAMD for distinguishing between patients with and without ALM in early-onset MDD. The area under the curve was 0.749. ROC: receiver operating characteristic. HAMD: Hamilton Rating Scale for Depression. MDD: major depressive disorder.

Based on the results of the univariate analysis in both groups, we performed a logistic regression model using patients with and without ALM as dependent variables and significant variables, including HAMD, HAMA, PANSS positive subscale, CGI, SBP, and TSH, as independent variables. We found that the HAMD total score (OR = 1.34, 95% CI = 1.18–1.53, *p* < 0.001) was the only risk factor for ALM in early-onset MDD patients. The risk of ALM increased by 34% for each point increase in the HAMD score. In addition, Figure 1 showed the AUC value of 0.749 for HAMD to differentiate patients with ALM from those without ALM (*p* < 0.001, 95% CI = 0.686–0.813) The cutoff value for HAMD was 29.5, the sensitivity was 0.643, and the specificity was 0.764 (Figure 1).

### 3.4. Risk Factors Associated with ALM in the Late-Onset Group

**Figure 2:**
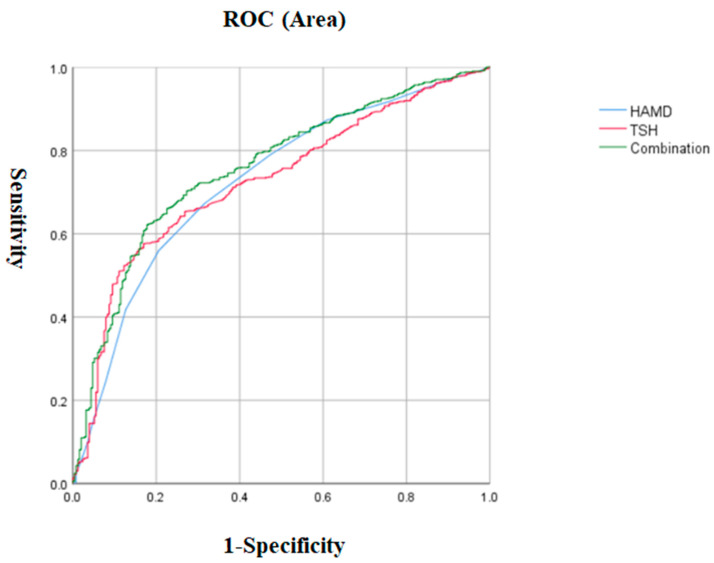
The discriminatory capacity of the HAMD score, TSH, and the combination of these two factors for distinguishing between patients with and without ALM in late-onset MDD. The area under the curve of the HAMD score, TSH, and the combination of these two factors were 0.76, 0.758, and 0.789, respectively. ROC: receiver operating characteristic. HAMD: Hamilton Rating Scale for Depression. TSH: thyroid-stimulating hormone. MDD: major depressive disorder.

We performed a logistic regression model using patients with and without ALM as dependent variables and significant variables in the univariate analysis, including disease duration, BMI, suicide attempts, anxiety, HAMD, PANSS positive subscale, CGI, SBP TSH, ATG, and blood glucose concentration, as independent variables. We found that the HAMD total score (OR = 1.19, 95% CI = 1.11–1.28, *p* < 0.001), TSH (OR = 1.25, 95% CI = 1.16–1.36, *p* < 0.001), CGI (OR = 1.7, 95% CI = 1.31–2.19, *p* < 0.001), and anxiety (OR = 2.22, 95% CI = 1.23–4.02, *p* = 0.008) were risk factors for ALM in patients with late-onset MDD. The risk of ALM increased by 19% for each point increase in the HAMD score, by 25% for each 1 mIU/L increase in the TSH concentration, and by 70% for each point increase in the CGI score. Patients with severe anxiety were 2.22 times more likely to develop ALM than those without severe anxiety. In addition, the AUCROC showed the following values for each risk factor: 0.531 for anxiety, 0.726 for TSH, 0.725 for HAMD, and 0.681 for CGI. The cutoff value for HAMD was 29.5, the sensitivity was 0.673, and the specificity was 0.684. The cutoff value for TSH was 503, the sensitivity was 0.576, and the specificity was 0.83. The cutoff value for CGI was 6.5, the sensitivity was 0.32, and the specificity was 0.941. The sensitivity for severe anxiety was 0.138, and the specificity was 0.925. Finally, when we combined the parameters with AUC values ≥ 0.7, we found that the combination of HAMD and TSH had a higher AUC value of 0.757 to distinguish patients with ALM from those without ALM (*p* < 0.001, 95% CI = 0.726–0.789) (Table 3 and Figure 2).

## 4. Discussion

To our knowledge, this is the first study focused on comparing the incidence and risk factors of ALM in Chinese outpatients with MDD at different ages of onset. Overall, this study showed the following: (1) the prevalence of ALM was higher in late-onset MDD patients than in early-onset MDD patients; (2) for both early-onset and late-onset MDD patients, those with comorbid ALM had higher scores on HAMD, HAMA, PANSS, and CGI; higher TSH levels, SBP, and DBP; and higher rates of psychotic symptoms compared to those without ALM; however, only in late-onset MDD, patients with comorbid ALM had higher rates of suicide attempts, anxiety, higher age, age at onset, and duration of illness, as well as greater levels of BMI, blood glucose, and A-TG compared to those without ALM; and (3) for early-onset MDD patients, only HAMD was significantly associated with ALM, whereas for late-onset MDD patients, HAMD, CGI scores, TSH levels, and anxiety were independently associated with ALM.

Our study found that late-onset MDD patients had a higher rate of ALM than early-onset MDD. Consistent with our study, Hickie et al. also found a higher vascular risk in patients with late-onset depression, showing 82% in patients with late onset and 57% in patients with early onset [36]. However, accounting for the cohort’s limited demographic (Han Chinese outpatients), these findings need replication in diverse populations and clinical settings. A 9-year study of a Swedish population sample indicated a strong association between the lipoprotein E (APOE) ε4 allele and late-life depression [37]. The lipoprotein E (ApoE) gene is a major genetic risk factor for late-onset psychiatric disorders. Smith et al. found that in middle-aged and older adults, higher levels of endothelial mesothelium thickness were associated with later depressive episodes [38]. It is known that lipid parameters are strongly associated with endothelial thickness [39,40]. Mulvahill et al. revealed that in older adults with depression, the presence of metabolic syndrome (MetS) was associated with more severe depressive symptoms, with a greater MetS component and lower HDL cholesterol having a comparable impact [41]. A meta-analysis of 29 studies conducted by Bharti et al. showed that patients with MDD had higher triglyceride and TG levels but lower TC and low-density lipoprotein compared to healthy individuals [42]. Based on the above studies, it is not difficult to explain the higher rate of ALM in late-onset MDD. Although these findings provide strong evidence for an association between late-onset MDD and ALM, studies reporting the association between early-onset MDD and ALM are still limited. Therefore, further studies are needed to elucidate the mechanisms of ALM in MDD patients of different age onset.

This study identified that the HAMD score was the only significant risk factor for early-onset MDD combined with ALM. The HAMD score is a clinical tool used to assess the severity of depression. Previous research has demonstrated a correlation between HAMD scores and lipid levels [43]. Variables such as anxiety lost significance after applying the Bonferroni correction, likely due to the inclusion of numerous variables that have less explanatory power regarding ALM than the HAMD score. We hypothesize that patients with early-onset MDD may experience a prolonged illness duration and more severe clinical symptoms, making the depression score more sensitive to lipid metabolic abnormalities. However, it is important to acknowledge that the relatively small sample size of 349 early-onset patients may also contribute to the reduced predictive efficacy of other variables for ALM.

We found that TSH levels were a strong predictor of late-onset MDD combined with ALM. In patients with late-onset MDD, the risk of ALM increased by 25% for each 1 mIU/L increase in TSH concentration. This aligns with the findings of Wang et al., who also reported that elevated TSH levels were significantly linked to higher TC and LDL levels [44]. Thyroid hormones play a key role in lipid metabolism, and lipid status deteriorates as TSH levels rise [45]. We hypothesize that inflammation may mediate the relationship between TSH and ALM [46,47]. However, while clinical studies have established a correlation between TSH and ALM, the underlying mechanisms remain poorly understood and warrant further investigation in future research.

Several limitations should be considered in the current study. Firstly, all MDD patients were outpatients from a single general hospital in Taiyuan, which may limit the generalizability of the findings to other ethnic groups or clinical settings. Future research should replicate this study in more diverse populations to validate the results. Secondly, the present study used a cross-sectional design; therefore, the causal relationship between early- and late-onset MDD and ALM cannot be directly inferred. Further large dataset studies using a longitudinal design are needed to support the results of this study. Thirdly, we did not collect data on several factors known to influence lipid metabolism, such as diet, physical activity, lipid-lowering medications, lifestyle, and socioeconomic status. Altered lipid metabolism (ALM) is a complex phenomenon that may be influenced by a range of clinical and potentially unidentified biological factors. Nevertheless, the factors we investigated were limited, including the exclusion of markers commonly associated with MDD, such as oxidative stress and immunological indicators. Fourthly, no information is provided regarding the subtype of depression (or the comorbidities) of depressed patients in this study. More studies are necessary to further explore differences in early-onset and late-onset MDD comorbid ALM between different depressive subtypes. Last but not least, thresholds for early-onset or late-onset MDD may lead to different outcomes, and standardizing the criteria in the future is necessary.

In conclusion, this study showed that the prevalence of ALM was significantly higher in late-onset (81.5%) than in early-onset MDD patients (20.6%). For early-onset MDD patients, the risk factor for combined ALM in MDD patients was HAMD; however, for late-onset MDD patients, ALM was significantly associated with higher HAMD, CGI scores, TSH levels, and anxiety. Due to the single-center design, cross-sectional nature, and absence of lifestyle data, our findings need to be carefully interpreted, and the results should be confirmed in future prospective trials.

## Figures and Tables

**Table 1:** Comparison of baseline variables between early-onset and late-onset MDD with ALM.

	Early Onset (*n* = 349)				Late Onset (*n* = 1369)			
	With ALM (*n* = 277)	Without ALM (*n* = 72)	F	*p*	With ALM (1116)	Without ALM (253)	F	*p*
Age, year	19 (18, 20)	19 (18, 20)	−0.262	0.793	39 (30, 48)	37 (29, 46.5)	−2.08	0.038
Duration of illness	3.5 (2.5, 5)	3.5 (2.5, 6)	−1.096	0.273	6 (3, 9)	4.5 (3, 7.5)	−3.949	<0.001
Age of onset	19 (18, 20)	19 (18, 20)	−0.67	0.503	39 (30, 48)	37 (29, 46)	−2.03	0.042
Sex, *n* (%)			0.76	0.383			0.545	0.46
1	115 (41.5)	34 (47.2)			353 (31.6)	86 (34)		
2	162 (58.5)	38 (52.8)			763 (68.4)	167 (66)		
BMI, kg/m^2^	24.17 (2.16)	24.03 (1.97)	0.244	0.622	24.5 (1.91)	24.08 (1.64)	10.739	0.001
Education, *n* (%)			3.936	0.268			1.691	0.639
1	9 (3.2)	1 (1.4)			333 (29.8)	70 (27.7)		
2	187 (67.5)	49 (68.1)			428 (38.4)	96 (37.9)		
3	76 (27.4)	18 (25)			282 (25.3)	73 (28.9)		
4	5 (1.8)	4 (5.6)			73 (6.5)	14 (5.5)		
Married, *n* (%)	25 (9)	7 (9.7)	0	1	974 (87.3)	210 (83)	2.865	0.091

Data expressed as mean ± SD, median (interquartile range), or percentage. Education degree: 1, middle school; 2, high school; 3, college; 4, graduate. BMI: body mass index.

**Table 2:** Comparison of clinical variables between early-onset and late-onset MDD with ALM.

	Early Onset (*n* = 349)				Late Onset (*n* = 1369)			
	With ALM (*n* = 277)	Without ALM (*n* = 72)	F	*p*	With ALM (1116)	Without ALM (253)	F	*p*
Suicide attempt, *n* (%)	48 (17.3)	7 (9.7)	1.95	0.163	254 (22.8)	37 (14.6)	7.676	0.006
Severe anxiety, *n* (%)	26 (9.4)	5 (6.9)	0.173	0.677	154 (13.8)	19 (7.5)	6.831	0.009
Exhibiting psychotic symptoms, *n* (%)	34 (12.3)	2 (2.8)	4.592	0.032	124 (11.1)	11 (4.3)	9.866	0.002
TSH, mIU/L	4.77 (3.2, 6.8)	3.76 (2.13, 4.51)	−4.793	<0.001	5.82 (2.44)	3.45 (1.87)	339.477	<0.001
FT3, pmol/L	4.97 (0.72)	4.88 (0.75)	0.995	0.319	4.91 (0.73)	4.81 (0.7)	3.676	0.055
FT4, pmol/L	16.65 (3)	17.05 (3.05)	0.996	0.319	16.73 (3.08)	16.54 (3.26)	0.741	0.389
Fasting plasma glucose, mmol/L	5.38 (0.7)	5.21 (0.5)	3.572	0.06	5.47 (0.63)	5.18 (0.61)	43.556	<0.001
LDL-C, mmol/L	3.01 (0.93)	2.3 (0.51)	38.969	<0.001	3.12 (0.87)	2.52 (0.48)	114.088	<0.001
HAMD	31 (28, 32)	28 (26, 29)	−6.557	<0.001	31 (29, 32)	28 (27, 30)	−11.271	<0.001
HAMA	21 (18, 23)	19 (17, 22)	−2.917	0.004	21 (19, 23)	19 (17, 22)	−5.834	<0.001
PANSS	7 (7, 9)	7 (7, 7)	−3.775	<0.001	7 (7, 9)	7 (7, 7)	−6.595	<0.001
CGI	6 (5, 7)	5 (5,6)	−4.566	<0.001	6 (5, 7)	5 (5, 6)	−9.642	<0.001
A-TG, IU/mL	19.12 (13.7, 31.29)	18.92 (13.22, 24.48)	−1.044	0.297	22.21 (15.02, 59.06)	19.89 (14.04, 33.65)	−2.248	0.025
A-TPO, IU/mL	17.13 (12.2, 33.06)	16.01 (11.2, 28.0)	−1.453	0.146	17.79 (12.32, 36.93)	17.48 (12.6, 28.81)	−0.867	0.386
TC, mmol/L	5.32 (1.14)	4.13 (0.63)	−14.012	<0.001	5.51 (1.06)	4.34 (0.56)	291.031	<0.001
HDL-C, mmol/L	1.25 (0.98, 1.42)	1.3 (1.18, 1.54)	−3.329	0.001	1.21 (0.95, 1.38)	1.27 (1.18, 1.52)	−6.840	<0.001
TG, mmol/L	2.34 (1.72, 2.92)	1.26 (1.09, 1.50	−10.193	<0.001	2.25 (1.7, 2.87)	1.26 (1.09, 1.47)	−19.775	<0.001
SBP, mmHg	110.9 (9.8)	106.1 (7.95)	14.529	<0.001	122.66 (9.73)	118.67 (9.63)	34.778	<0.001
DBP, mmHg	72.5 (6.14)	70.36 (5.37)	7.291	0.007	77.26 (6.63)	75.55 (6.02)	14.222	<0.001

Data expressed as mean ± SD, median (interquartile range), or percentage. TSH: thyroid-stimulating hormone. FT3: free triiodothyronine. TF4: free thyroxine. LDL-C: low-density lipoprotein cholesterol. HAMD: Hamilton Rating Scale for Depression. HAMA: Hamilton Anxiety Scale. PANSS: Positive and Negative Syndrome Scale. CGI: clinical global impression. A-TG: anti-thyroglobulin. A-TPO: thyroid peroxidase antibody. TC: total cholesterol. HDL-C: high-density lipoprotein cholesterol. TG: triacylglycerols. SBP: systolic blood pressure. DBP: diastolic blood pressure.

**Table 3:** The risk factors of ALM in patients with late-onset MDD.

	B	Wald	*p*	OR	95% CI Lower	95% CI Upper
Suicide attempt	−0.391	3.001	0.083	0.676	0.434	1.053
HAMD	0.174	24.247	<0.001	1.19	1.11	1.275
TSH	0.226	31.344	<0.001	1.254	1.158	1.357
CGI	0.528	16.475	<0.001	1.695	1.314	2.187
Fasting plasma glucose	0.265	3.648	0.056	1.303	0.993	1.711
Severe anxiety	0.799	6.953	0.008	2.223	1.228	4.024

Dependent variable: ALM; independent variables: suicide attempt, HAMD, TSH, CGI, fasting plasma glucose, and severe anxiety. HAMD: Hamilton Rating Scale for Depression. TSH: thyroid-stimulating hormone. CGI: clinical global impression.

## Data Availability

The original contributions presented in the study are included in the article, further inquiries can be directed to the corresponding authors.
